# Applying ischemic preconditioning prior to endurance training improves hematological profile and performance in long-distance runners

**DOI:** 10.1007/s00421-025-06120-6

**Published:** 2026-01-16

**Authors:** Ioannis Loukas, Dimitrios Stergiopoulos, Alexandros Sotiridis, Maria Koskolou, Nickos Geladas

**Affiliations:** Division of Sports Medicine and Biology of Exercise, Faculty of Physical Education and Sport Science, National and Kapodistrian University of Athens, Ethnikis Antistasis 41, Daphne, Athens, 17237 Greece

**Keywords:** Training methods, Hematological adaptations, Endurance performance

## Abstract

**Purpose:**

Ιschemic preconditioning is well-accepted to improve exercise capacity and performance. The purpose of this study was to examine whether ischemic preconditioning performed prior to endurance training in a long-term fashion enhances physiological adaptations and performance.

**Methods:**

Sixteen male distance runners (age: 34.1 ± 5.1 yrs, VO_2_max: 55.0 ± 2.0 ml/kg/min) participated in the study. Training consisted of two high-intensity interval sessions (90–100% VO_2_max) and three continuous sessions (70–80% VO_2_max) per week for eight weeks. Participants were divided into two groups of similar fitness level. Before interval training the ischemic preconditioning group (ISC, *n* = 8) underwent 3 × 5 min total blood flow occlusion in each leg (applied external pressure using cuffs: 250mmHg), while the control group (CON, *n* = 8) underwent the same protocol but without pressure being applied. Pre and post training VO_2_max, hematological profile, and blood pessure was evaluated, while a field-specific test of 5 × 1000 m with 2 min break was executed.

**Results:**

Training increased VO_2_max (*p* < 0.01) in both groups (ISC: 3.92 ± 0.1 vs. 4.22 ± 0.1 L/min, CON: 3.94 ± 0.20 vs. 4.05 ± 0.19 L/min) but the increase was higher in ISC (training group x time interaction *p* = 0.001). Post training average running time during the 5 × 1000 m test was faster in ISC than CON (ISC: 211 ± 3 vs. 200 ± 4 s, CON: 210 ± 4 vs. 205 ± 5 s) (training group x time interaction *p* = 0.020). Pre versus post values in ISC compared to CON showed a more pronounced increase in blood volume (ISC: 4887 ± 448 vs. 5415 ± 438 ml, CON: 4788 ± 489 vs. 5103 ± 517 ml) (training group x time interaction *p* = 0.012) and plasma volume (ISC: 2663 ± 309 vs. 3114 ± 271 ml, CON: 2620 ± 306 vs. 2912 ± 246 ml) (training group x time interaction *p* = 0.009). Moreover, there was a training effect (*p* < 0.05) for hemoglobin mass and red cell volume without differences between groups.

**Conclusions:**

The intervention of ischemic preconditioning prior to interval training may enhance physiological adaptations mainly through hematological alterations and, thus, improve athletic performance.

**Supplementary Information:**

The online version contains supplementary material available at 10.1007/s00421-025-06120-6.

## Introduction

Long distance running is a popular and demanding endurance activity. Maximal oxygen consumption (VO_2_max), lactate threshold, running economy (oxygen consumption at a given workload) and physiological resilience are determinant factors of endurance running performance (Jones and Kirby 2025; Unhjem 2024) and their development depends on the intensity of training stimulus. Various methods are currently used to optimize performance such as high-intensity interval training (HIIT), altitude training, heat acclimation and training with low glycogen stores (Oberholzer et al. 2019; Ronnestad et al. 2022; Schmidt and Prommer 2008; Hulston et al. 2010; Van Proeyen et al. 2011). However, training at altitude can be insufficient to enhance performance more than traditional training at sea-level as a recent meta-analysis showed (Dorelli et al. 2025). Ηeat acclimatization strategies appear to be beneficial for performance and a favorable hematological profile, namely increasing hemoglobin mass (HBmass) (Oberholzer et al. 2019; Nybo et al. 2022). In summary, there is a need for new effective training modes to be developed and to enhance performance.

Ischemic preconditioning refers to a maneuver consisting of temporarily occluding blood flow of a tissue. It was first developed to enhance organ survival upon exposure to ischemia-reperfusion of a tissue for medical reasons but its application expanded in sports and exercise. During occlusion, the tissue starves for oxygen leading to an increase of vasodilatory substances like NO, ATP and bradykinin, resulting in a sharp increase of blood flow when occlusion ceases, a phenomenon called reactive hyperemia (Kimura et al. 2007). Ιschemic preconditioning performed prior to muscular work has been claimed to enhance aerobic metabolism, maximal power output and performance (Kido et al. 2015; Crisafulli et al. 2011; Jean-St-Mitchel et al. 2011; Groot et al. 2010; Paul and Van Guilder 2019; Paradis-Deschenes et al. 2018; Griffin et al. 2018). Moreover, various studies using meta-analysis on the effects of ischemic preconditioning in exercise performance show positive results and performance enhancement (Chen et al. 2025; Zhou et al. 2025; Incognito et al. 2016; Salvador et al. 2016).

During running exercise, ischemic preconditoning reduced lactate accumulation in submaximal intensities improving subsequent 5 km running time-trial performance (Bailey et al. 2012). Interestingly, a recent study demonstrated that ischemic preconditioning can enhance performance via neural mechanisms augmenting neural drive to the exercising muscles and improving muscular force (Cruz et al. 2024). That means that fatigue tolerance, anaerobic capacity, or muscular efficiency might be increased.

Keeping this in mind, training with regular ischemic preconditioning would let athletes achieve higher running velocities, inducing greater adaptations and improving performance. That was not the case, however, in one study with endurance runners which showed that training for two months with ischemic preconditioning applied prior training did not improve performance (Slysz and Burr 2019). However, this study had a counter intuitive limitation that trainers did not readjust running velocities to a higher speed in the intervention group, as ischemic preconditioning increases peak power output. To our understanding, the athletes running with the same velocities as before, after ischemic preconditioning would train with lower stimulus expressed as percentage of maximal aerobic velocity. In contrast, a recent study in swimming showed that 8-weeks of training with ischemic preconditioning improved performance via improved power output and delaying fatigue (Yang et al. 2025).

Endurance training elicits adaptations of central and peripheral origin. Indeed, increases in intravascular blood volumes and HBmass would enhance oxygen-carrying capacity and act to enhance aerobic performance (Montero et al. 2017). The superposition of the ischemic stimulus might serve to further increase plasma volume. A former study showed that reduction in central venous pressure in an acute manner can augment erythropoeisis through increases in volume-regulating hormones (Montero et al. 2016). Ischemic preconditioning could probably produce a similar effect decreasing central venous pressure during occlusion since the trapped blood to the tissues below the cuff could decrease venous return as two studies reported a decreased stroke volume during occlusion (Cherouveim et al. 2023; Ozaki et al. 2010), and in the long-term might stimulate an increase in plasma and blood volume. However, no clear evidence exists to support this assumption. A hypervolemia-driven erythropoiesis would eventually occur so that the haematocrit is homeostatically regulated albeit at higher total HBmass and blood volume (Montero et al. 2016).

The impact of reaching maximal arterial blood pressure and its role on performance remains unexplored. It is assumed that an elevated systolic pressure would enhance blood perfusion to the working muscle increasing blood flow as long as functional sympatholysis is not impaired (Calbet and Joyner 2010). Indeed, in a recent study where sympatholysis was compromised by blood flow occlusion, it was found that each exercising individual reaching prematurely his maximal systolic blood pressure led to cessation of exercise, and thus, limiting performance (Cherouveim et al. 2023). However, the long-term suprasystolic effect induced by ischemic preconditioning on blood pressure and its probable effect on performance is currently unknown.

The purpose of this study was to investigate whether the application of cuff pressure on the thighs causing blood flow occlusion prior to HIIT will enhance physiological adaptations and subsequently improve endurance running performance. We supposed that repetitive occlusion and hyperemia via ischemic preconditioning when used in a long-term fashion prior to HIIT could enhance hematological adaptations and blood perfusion through increased systolic blood pressure, thus, improving running performance.

## Methods

Initially, Eighteen male long-distance runners volunteered to participate in the study. All participants were healthy, and non-smokers. They were all informed about the training protocol, experimental procedures, and the possible risks involved and provided signed consent. Two participants dropped out, one at the very beginning after completion of the initial tests at the first week due to personal reasons, and the second one after the second week of training due to a comeback of a treated injury. Both of them were replaced so as the two groups finally constituted of 8 participants. The study was approved by the University’s Ethical Committee for human experimentation and conformed to the principles of the Declaration of Helsinki (Number: 1564).

### Experimental design

**Fig. 1:**
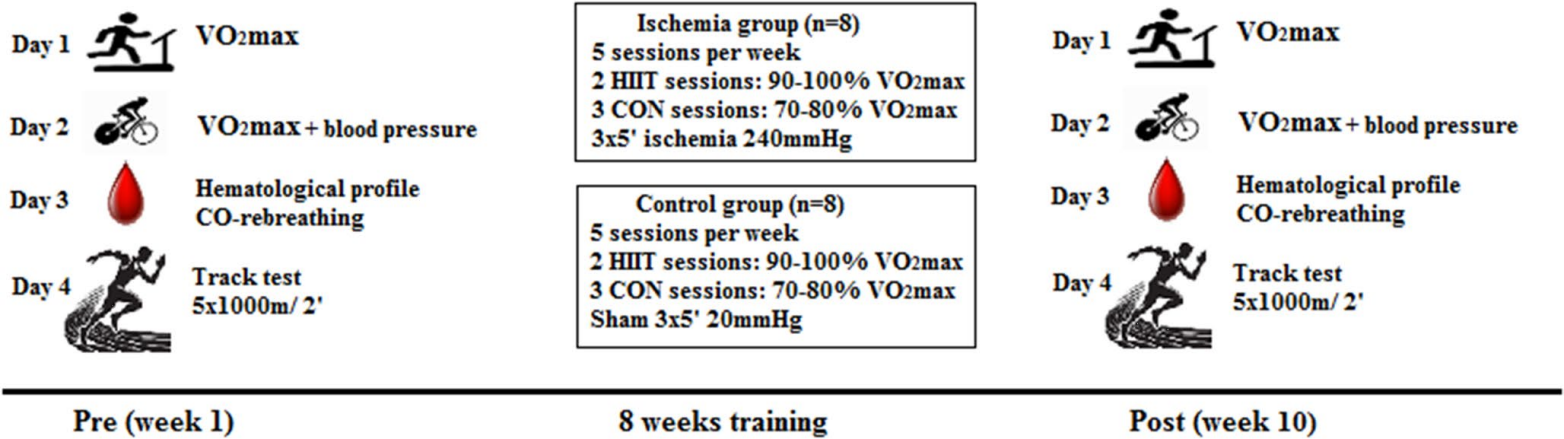
Study design with experimental protocol and training intervention (*n* = 16). Day 1: VO_2_max test in a treadmill ergometer. Day 2: VO_2_max test in a cycle ergometer with blood pressure response recording. Day 3: Hematological profile assessment with CO-rebreathing method. Day 4: Performance test at a track executing 5 repetitions of 1000 m with 2 min passive rest. Training intervention: Endurance training 5 times per week for 8 weeks in two different groups (ISC and CON). Two sessions included high-intensity interval training and three sessions comprised of moderate-intensity continuous training

A battery of tests before and following the 8-week training protocol was used to assess physiological, hematological and performance parameters of the indviduals. Specifically, measures for VO_2_max, blood pressure and hematological variables were performed in the lab whereas running performance was measured in an outdoor track. There was a 48-h break between each evaluation and there were completed within one week. The study took place between January and April under mild environmental conditions (15–25 °C). The detailed experimental protocol and training intervention is presented in Fig. 1.

### VO_2_max test

A treadmill VO_2_max test was performed by the participants to assess maximal aerobic capacity and velocity (vVO_2_max), ventilatory thresholds as well as to identify training zones (subsequently used for the training sessions). Participants were asked to refrain from caffeine or any ergogenic supplement the day of testing. Initially, anthropometric measurements for weight, height and body composition were assessed. Body fat was calculated with skinfold measurement of 7 sites for body density (Jackson and Pollock 1985) and using the equation of Siri (1961) for the percentage of body fat. Following a typical warm-up protocol treadmill was set at a 1% incline and initial velocity was 8 km/h and was increased 1 km/h every 1 min. All tests were performed until volitional exhaustion. Standard criteria were used to confirm the attainment of VO_2_max: (a) VO_2_ plateau despite increases in treadmill speed, (b) RER values > 1.15, (c) HRmax > 90% age predicted value. During the whole testing gas exchange was recorded breath-by-breath via an open-circuit spirometry (Ultima CPX, MedGraphics, USA). Before each test, the gas analyzers for O_2_ and CO_2_ and the pneumotachograph were calibrated with two different gas mixtures (a) 12% O_2_ and 5% CO^2^ balanced in N_2_ and (b) 21% O_2_ and 0.01% CO_2_ balanced in N_2_ and with a 3-L syringe (Ultima CPX, MedGraphics, USA), respectively. The heart rate during the test was recorded telemetrically (Polar V-800, Finland).

VO_2_max was calculated as the average oxygen consumption of the last 20 s of the final stage with the prerequisite that a plateau was not reached in the previous stage. Maximal aerobic velocity was the speed corresponding to VO_2_max and was calculated with the equation of Kuipers et al. (1985): vVO_2_max = Scom + (t/ΔΤ) x ΔS,

where vVO_2_max is the maximal aerobic velocity, Scom is the last successfully completed stage, t is the time of the last unsuccessful completed stage, ΔΤ is the duration of every stage and ΔS is the increase in velocity at every stage. If a plateau on VO_2_ was reached at a previous stage before exhaustion, the last stage was excluded from the equation and vVO_2_max was at the speed that VO_2_max was reached.

First and second ventilatory thresholds were assessed using breaking points for end-tidal partial pressures and ventilatory equivalents for O_2_ and CO_2_. Specifically, first ventilatory threshold was determined at the point of: (a) nadir or first increase of V_E_/VO_2_ versus workload without a simultaneous increase in V_E_/VCO_2_ versus workload, (b) nadir or first rise of PETO_2_, while PETCO_2_ remains constant or is increasing. Second ventilatory threshold was determined at the point of: (a) inflection of V_E_ versus VCO_2_, (b) nadir or nonlinear increase of V_E_/VCO_2_ versus workload, (c) deflection point of the end tidal PETCO_2_ (Beaver et al. 1986).

### Blood pressure variables

An incremental cycling test to exhaustion was performed to measure peak systolic blood pressure with the noninvasive method of photoplethysmometry (Finapres, FMS, The Netherlands) with the cuff attached on the left middle finger. The Finapres traces the finger pulse waveform to reconstruct aortic flow and calculate arterial pressure. To minimize artifacts and measurement’s noise, the left hand was relaxing and immobilized on a stable board so as not to put discomfort while exercising. The hand was placed at heart level and with the fingers warmed prior to measurement and a small towel was used to cover the hand to keep it warm during exercise. Room temperature was between 22 and 24 °C. Participants refrained from caffeine and supplements the day of testing. Before exercise, the participants sat quietly without any annoyance to record resting blood pressure. The measurement lasted for 5 min in which no verbal communication was held and without visual access to the blood pressure values on the screen. After that, participants sat on a constant-load cycle ergometer (Lode, Groningen, Netherlands), and exercise began with initial intensity at 30 W and 30 W increases per minute until volitional exhaustion. Cadence was kept between 70 and 90 rpm and when cadence fell to 50 rpm exercise was terminated. Gas exchange was recorded as Sect. 2.2. Blood pressure values at rest were calculated as the average value of the last 20 s of each minute. Peak blood pressures values were calculated as the average value of the last 20 s of the final stage.

### Hematological variables

Participants came to the lab in an euhydrated state. The carbon monoxide (CO)-rebreathing method was performed using a fully automated instrument (OpCo; Detalo Instruments, Horsholm, Denmark). Initially, participants rested in a legs-up supine position for 10-min before the onset of the procedure. After that, participants remained in the same position and were connected to a CO pulse oximetry sensor (Rad-57, Masimo Corporation, Irvine, CA, USA). Duplicate finger capillary blood samples were drawn to determine hematocrit (Hct) and [Hb] levels. Hct values were determined using a standard Hct scale (Haematocrit Reader, Hawksley Inc., UK) after a 5-min centrifugation of blood at 4000 rpm (Micro Haematocrit Mk5 Centrifuge, Hawksley, UK). [Hb] was assessed photometrically using an automatic analyzer (Diaspect TM, EKF Life Sciences, Germany). After the breathing circuit was flushed with 100% O_2_, the participants were breathing the pure gas for two minutes to reduce the amount of nitrogen that would later be exhaled into the rebreathing circuit. Thereafter, the participants were switched to the rebreathing circuit and a bolus corresponding to 1.0 ml kg^−1^ body weight of chemically pure CO (99.997%, Linde Hellas, Greece) was administered into the closed rebreathing circuit. The participants then completed 6 min of CO rebreathing and inhaled room air for the remainder of the 10-min session. A plateau in SpCO was confirmed by virtue of the maximal SpCO value being noted before the end of the session. If that was not the case, the session was extended for two minutes so that no further increase in SpCO would be recorded. The only participant who felt discomfort earlier than the six minutes period was switched to room air at exact four minutes of CO rebreathing and the resultant ΔSpCO was included in the analysis. O_2_ was provided to the participant on a demand basis. Individuals inhaled room air for the remainder of the 10-min session. The remaining CO in the system was measured by a pre-calibrated CO sensor (Draeger Pac 6000/6500, Lübeck, Germany) to determine the eventual dose not inhaled. According to ΔHbCO, [Hb] and Hct, total blood volume (BV), plasma volume (PV), red blood cell volume (RBCV) and hemoglobin mass (Hbmass) were determined (Siebenmann et al. 2017a, b). The value of SpCO was recorded every minute throughout the rebreathing session. The highest SpCO value recorded during the rebreathing session as well as the time when this occurred were noted. ΔCOHb was calculated as the difference between the highest and the lowest SpCO value. The non-invasive determination of hemoglobin mass using a pulse CO-oximeter has been shown to be accurate for individuals possessing hemoglobin mass and total blood volumes lower than 905 g and 6153 mL, respectively (Fagoni et al. 2018). Even though duplicate measurements would reduce the typical error, an upper limit of a single CO inhalation allowed (even for diagnostic purposes) as it was recently decided by UCI and was similar to what the individuals experienced in the present study.

### Field test

A specific running test in the field with repeated intervals took place to assess running performance. Warm-up was the same as in training (see training intervention section), and then participants ran 5 × 1000 m interspersed by 2 min rest periods, and were asked to regulate their power output through intervals so as to complete the test with the fastest possible time. This test is considered as a closed-loop test which allows the participants to self-regulate their power output to exhaustion. Furthermore, this test is performed approximately at 95% VO_2_max as previous studies showed (Seiler and Sjursen 2004; Santos-Concejero et al. 2017) i.e. at near-maximal aerobic intensity and can successfully assess aerobic capacity. The test was performed in an Olympic size track (400 m) in the afternoon (19:00–21:00 p.m.) under the same environmental conditions (15–20 °C).

### Training intervention

Running training lasted 8 weeks. Participants were split into two groups (*n* = 8 each group), one group served as the control group (CON) that performed endurance training and the other group additionally underwent the muscular ischemia intervention (ISC). Both groups were matched in terms of anthropometric traits (CON: height 175 ± 3 cm, weight 70.5 ± 4.9 kg, ISC: height 178 ± 4 cm, weight 71.2 ± 3.3 kg), and fitness level (CON VO_2_max: 55.0 ± 2.0 ml/kg/min, ISC VO_2_max: 55.3 ± 1.2 ml/kg/min). Muscular ischemia was induced by applying suprasystolic pressure (250 mmHg) using a pair of inflated cuffs (Kessler KS106, Germany, length: 98 cm, width: 14 cm) on the subjects’ thighs as close to the pelvis as possible. Training frequency was 5 times per week, with 2 HIIT (90–100% VO_2_max) sessions interspersed by 3 continuous training sessions at an intensity 5–10% below the second ventilatory threshold i.e. at ~ 70–80% VO_2_max. The two HIIT sessions were separated by 2 low intensity training sessions within a week. Before HIIT training session the ischemia group performed 3 repetitions of 5 min duration each one bilateral total blood flow occlusion to the working muscles using an external cuff application reaching 250 mmHg. The blood flow occlusion-induced hyperemia has beneficial effect on performance lasting for 90 min (Bailey et al. 2012). As a result, training had to be completed within this time frame following the ischemic maneuver. Subsequently, after the completion of the ischemic maneuver the participants started warm-up right away for 25–30 min (15 min easy self-pacing running, 5–10 min dynamic stretching and 6 strides). The warm-up protocol was the same for all HIIT sessions. HIIT lasted about 30 ± 5 min in total (break time between running bouts was included) and then 5–10 min of cool-down running was performed. Control group completed the same protocol and training with ISC group using cuffs before HIIT with minimal external pressure. Single HIIT session consisted of bouts in the form of 15 times (x) 200 m or 10 × 300 m or 8 × 400 m corresponding to the 100% vVO_2_max with work to break ratio at 1:1 of passive rest, 6 × 800 m or 5 × 1000 m at 95–97% vVO_2_max with 2 min passive rest, 4 × 2000 m or 3 × 3000 m at 90–92% vVO_2_max with 3 min passive rest. One HIIT of the week was at 100% vVO_2_max and the other HIIT was alternated of 95–97% vVO_2_max and 90–92% vVO_2_max between weeks. Each HIIT training session was separated by 48–72 h to another one.

Continuous training (CON) consisted of three training sessions per week with 2 sessions serving as recovery runs lasting for 45–60 min and one long run at the weekend lasting up to 150 min. Running pace was maintained at around the velocities corresponding to 70–80% VO_2_max and heart rate was also monitored. Long run sessions varied between 90 and 150 min depending on the periodization phase. Intensity and volume of continuous training was the same in both groups. Cuffs were not applied in both experimental groups prior continuous training as studies have shown that active hyperemia due to vascular occlusion had no effect at moderate exercise intensities (Kaur et al. 2016). The detailed training intervention is provided as a supplementary table at the end of the manuscript.

At the end of each training session, participants were asked to rate their RPE (Borg scale, 6–20). Training was guided and supervised by the same researcher (I.L.). Post-training measurements were performed within 4–6 days after last bout of HIIT. During the week of testing no HIIT session was performed for both groups and no ischemia applied to the ISC group. Each test was separated by 48-h break. In the day of testing no training session took place, while the next day participants were confined themselves to light recovery runs early in the afternoon with 14-18-h space from the next test.

### Statistical analysis

Training characteristics were analyzed with an independent samples t-test. For physiological variables, after normality criteria were confirmed, a two-way repeated measures mixed-design analysis of variance (training group x time) was used to explore main effects and interaction (training group x time) of the independent variables on several physiological, hematological and performance parameters. In case of significant main effect or interaction, a Tukey post-hoc test was employed to locate specific differences. Linear regressions were also used to determine the Pearson’s product-moment correlation coefficients (r) between changes in key physiological variables (hemoglobin mass and intravascular volumes) and post-protocol changes in aerobic exercise performance parameters i.e. VO_2_max, vVO_2_max, performance in 5 × 1000 m. Data were expressed as means ± standard deviation (SD). Statistical significance threshold was set at *p* < 0.05.

## Results

### Training intervention

Training did not induce any significant change in body composition for neither CON nor ISC group. Pre and post training body mass and fat for CON was 70.5 ± 4.9 vs. 69.5 ± 4.5 kg and 11.0 ± 3.9 vs. 10.2 ± 3.6%, respectively. For ISC, pre to post training weight and body fat was 71.2 ± 3.3 vs. 70.0 ± 3.2 kg and 11.1 ± 3.5 vs. 9.8 ± 3.3%, respectively. No differences were also recorded between groups (*p* > 0.05) (Table 1). Total training volume and training volume in continuous sessions, expressed as distance covered per week, did not differ (*p* > 0.05) between groups (CON: 80.6 ± 4.0 vs. ISC: 80.2 ± 3.0 km and CON: 66.3 ± 4.0 vs. ISC: 66.1 ± 3.0 km), respectively. Heart rate and RPE in continuous training were similar for both groups (CON: 156 ± 12 vs. ISC: 152 ± 11 bpm and 11.2 ± 1.5 vs. 10.7 ± 0.9 at the Borg scale for CON and ISC respectively) (*p* > 0.05). HIIT sessions had an identical total volume for both groups (114.4 ± 8 km). ISC achieved higher values of average training velocity than CON in HIIT sessions (18.0 ± 0.4 vs. 17.4 ± 0.3 km/h, + 3.3% *p* = 0.001). Accordingly, the ISC group completed total HIIT training volume at a significant shorter time than CON (389 ± 32 vs. 402 ± 24 min, *p* = 0.001). ISC had also a reduced HIIT training load than CON (802 ± 82 vs. 835 ± 77 RPE·min, *p* < 0.001). Heart rate and RPE in HIIT did not differ between groups. Results of training are summarized at Table 2.

### VO_2_max

**Fig. 2:**
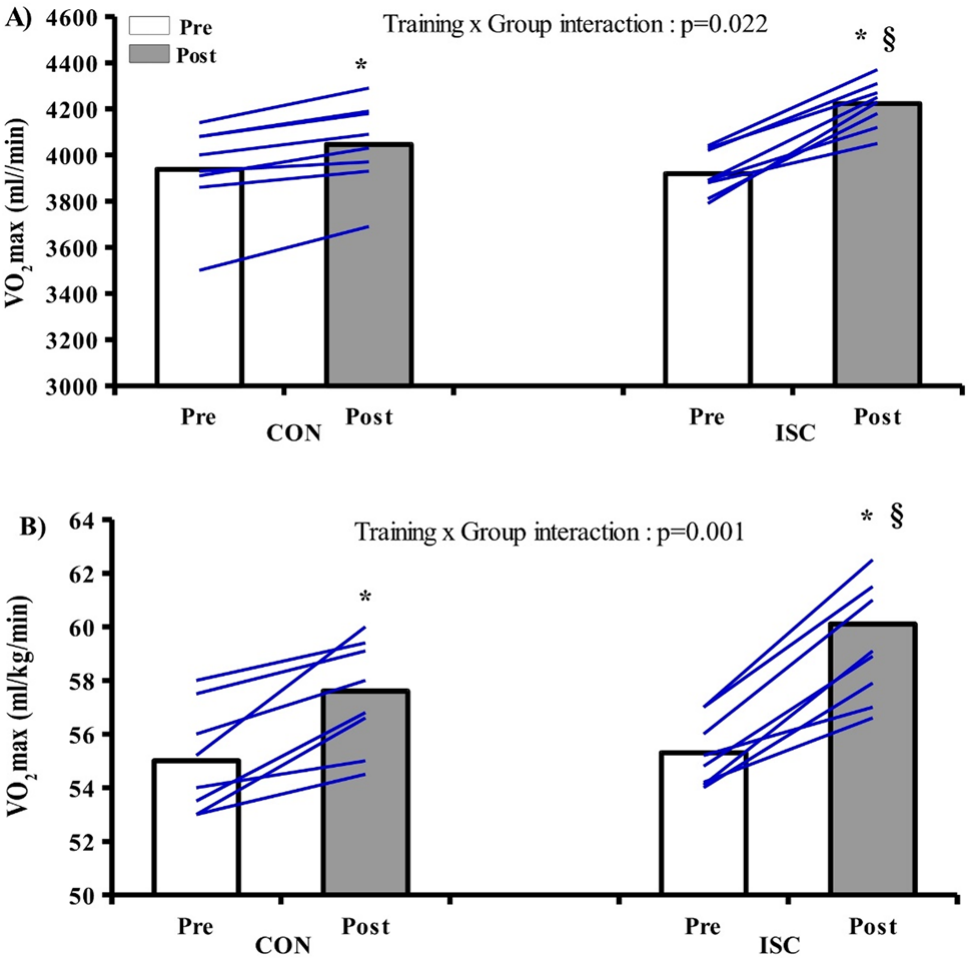
Individual and mean values of: VO_2_max **A** absolute values, **B** relative values for CON (*n* = 8) and ISC (*n* = 8) before and after 8 weeks of training. *significant difference pre vs. post. §significant difference between groups in post. *p* < 0.05

Training produced a significant increase in VO_2_max in both groups (ISC: 3.92 ± 0.10 vs. 4.22 ± 0.10 L/min, + 7.6%, CON: 3.94 ± 0.20 vs. 4.05 ± 0.19 L/min, + 2.7% *p* < 0.001), but the increase was higher in ISC (training group x time interaction *p* = 0.001) (Fig. 2). vVO_2_max also increased after training in both groups (ISC: 18.4 ± 0.9 vs. 19.6 ± 0.9 km/h, + 6.5%, CON: 18.2 ± 0.7 vs. 18.9 ± 0.6 km/h, + 3.8%, *p* < 0.001) with a higher increase observed in ISC (training group x time interaction: *p* = 0.048). There was also a training effect improving (*p* < 0.001) the running velocity and VO_2_ at the second ventilatory threshold but with no difference between groups, and a training effect (*p* = 0.004) of the percentage of VO_2_max corresponding to the second ventilatory threshold. Ergospirometric variables are presented in Table 3.

### Hematological variables

**Fig. 3:**
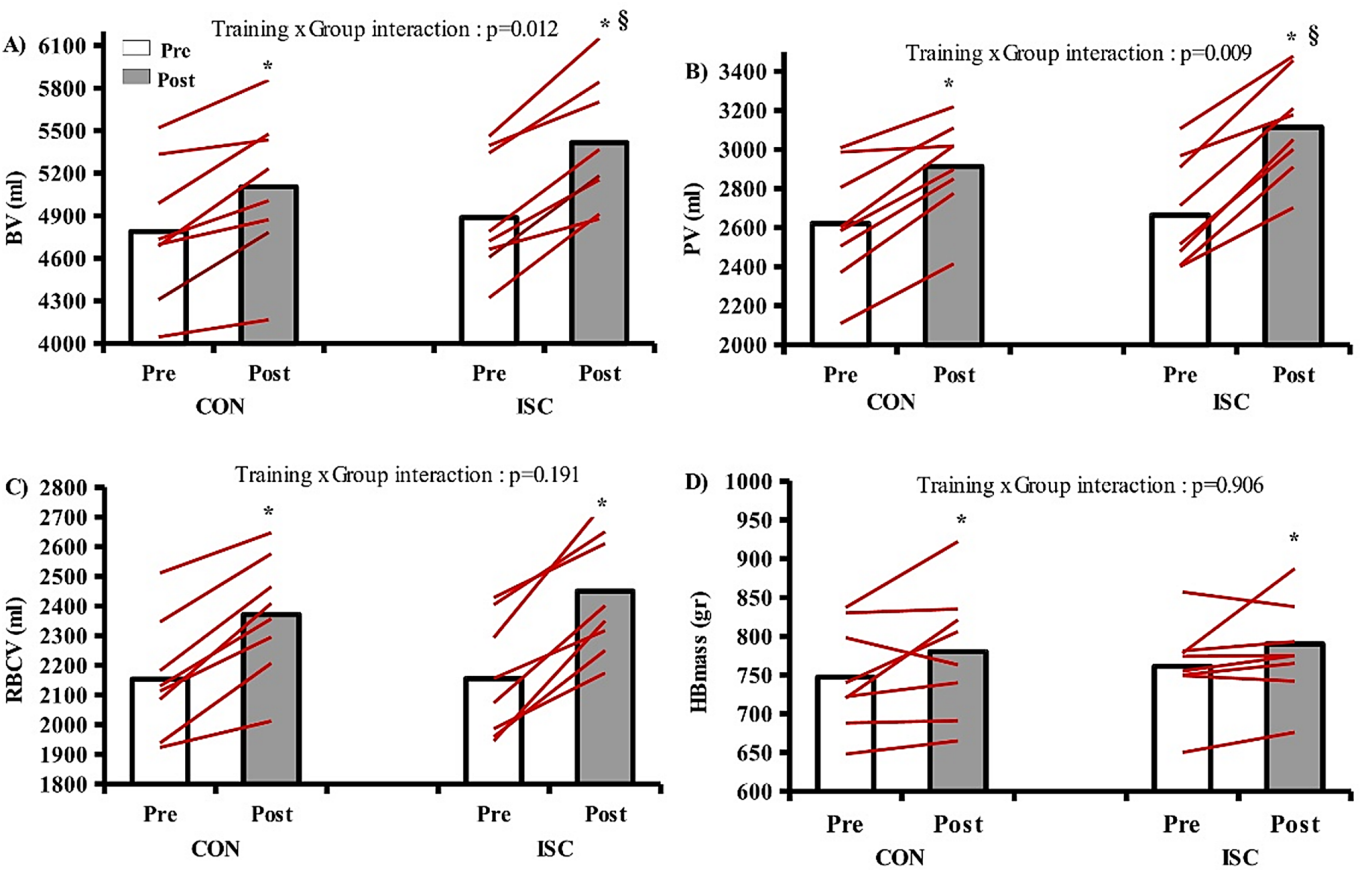
Individual and mean values of hematological variables pre and post for CON and ISC. **A** blood volume, **B** plasma volume, **C** red blood cell volume, **D** hemoglobin mass. *significant difference pre vs. post within groups. §significant difference between groups in post. *p* < 0.05

Training induced significant hematological adaptations (Fig. 3). Blood volume increased in both groups (training effect *p* < 0.001) but ISC had a more pronounced increase compared to CON (ISC: 4887 ± 448 vs. 5415 ± 438 ml, + 10.8%, CON: 4788 ± 489 vs. 5103 ± 517 ml, + 6.5%, training group x time interaction: *p* = 0.012). Plasma volume also increased significantly (*p* < 0.001) in both groups, but the increase was higher in ISC (ΙSC: 2663 ± 309 vs. 3114 ± 271 ml, + 16.9%, CON: 2620 ± 306 vs. 2912 ± 246, + 11.1%, group x time interaction: *p* = 0.009). There was a training effect for red cell blood volume but with no differences between groups (ISC: 2155 ± 197 vs. 2450 ± 230 ml, CON: 2153 ± 197 vs. 2371 ± 203 ml, *p* < 0.001). Hbmass increased after training across groups (ISC: 761 ± 70 vs. 790 ± 90 gr, CON: 747 ± 67 vs. 780 ± 87 gr, *p* = 0.046). Training did not induce any significant adaptations for hematocrit in both groups (ISC: 44.0 ± 2.6 vs. 45.2 ± 1.0%, CON: 44.9 ± 0.7 vs. 46.4 ± 1.3%). However, training induced a significant effect (*p* = 0.023) in ISC for Hb concentration and a significant interaction (*p* = 0.044) between groups (ISC: 16.2 ± 1.2 vs. 14.8 ± 0.8 g/dl, CON: 15.8 ± 1.2 vs. 15.6 ± 1.2 g/dl) (Table 1) (Fig. 3).

**Fig. 4:**
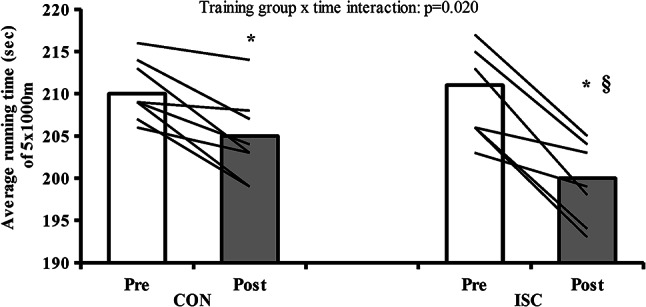
Individual average running times and mean values of performance in the running 5 × 1000 m test pre and post training in CON (*n* = 8) and ISC conditions (*n* = 8). *significant difference pre vs. post within groups. §significant interaction between groups after training. *p* < 0.05

### Blood pressure variables

**Fig. 5:**
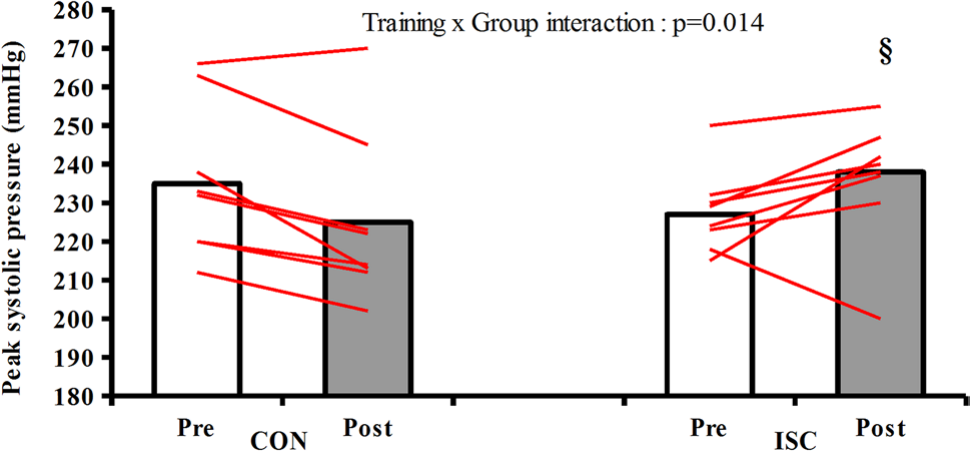
Individual and mean values of peak systolic blood pressure pre and post training in CON (*n* = 8) and ISC (*n* = 8) group.§signifacant interaction between groups after training. *p* < 0.05

Training had a significant effect (*p* < 0.001) reducing systolic blood pressure at rest for both ISC and CON without differences between groups (ISC: 124 ± 4 vs. 119 ± 5 mmHg, CON: 121 ± 3 vs. 117 ± 3 mmHg). There was also a training effect (*p* = 0.043) at rest with decreased mean arterial pressure for both groups (ISC: 92 ± 3 vs. 88 ± 3 mmHg, CON: 91 ± 2 vs. 87 ± 2 mmHg). Training did not induce any change in diastolic blood pressure (ISC: 70 ± 2 vs. 70 ± 2 mmHg, CON: 72 ± 2 vs. 69 ± 2 mmHg). During exercise an interaction effect (*p* = 0.014) between groups was seen for peak systolic arterial blood pressure with an increase in ISC group while a decrease recorded in CON (ISC: 227 ± 10 vs. 238 ± 20 mmHg, CON: 235 ± 19 vs. 225 ± 22 mmHg) (Fig. 5) as well for mean arterial pressure (ISC: 143 ± 5 vs. 148 ± 11 mmHg, CON: 145 ± 21 vs. 139 ± 21 mmHg, *p* = 0.007). Peak diastolic arterial blood pressure did not reach statistical significance between groups (interaction effect: *p* = 0.050, ISC: 95 ± 5 vs. 103 ± 12 mmHg, CON: 100 ± 12 vs. 97 ± 1 mmHg). Peak power output in the cycle ergometer increased in both groups after training but with a more pronounced increase (training group x time interaction: *p* = 0.031) in ISC group (ISC: 300 ± 10 vs. 315 ± 11 watts, CON: 295 ± 8 vs. 306 ± 9 watts, *p* = 0.005). VO_2_max in cycle ergometer increased significantly after training for both groups but without difference between them (ISC: 51.2 ± 1.5 vs. 54.6 ± 1.9 ml/kg/min, CON: 51.0 ± 2.1 vs. 54.0 ± 1.7 ml/kg/min, *p* < 0.001). Ergospirometric variables derived from the exhaustive test on the cycle ergometer are presented in Table 4.

### Field test

While there was no difference in performance at baseline between groups (Fig. 4), training induced a significant performance enhancement (*p* < 0.001) improving running bout times and velocity for both groups but with a more pronounced improvement in ISC group (ISC: +5.0% vs. CON: +2.5%, training group x time interaction: *p* = 0.020). Average running time for the five repetitions before and after training in CON was: 210 ± 4 vs. 205 ± 5 s (effect of training: *p* < 0.001), and in ISC was: 211 ± 3 vs. 200 ± 4 s (effect of training *p* < 0.001, training group x time interaction: *p* = 0.020). Average running velocity for the five repetitions before and after training in CON was: 17.1 ± 0.3 vs. 17.5 ± 0.4 km/h (effect of training: *p* < 0.001), and in ISC was: 17.0 ± 0.4 vs. 18.0 ± 0.6 km/h (effect of training *p* < 0.001, training group x time interaction: *p* = 0.019).

### Correlation analysis

High correlations were noted between changes in VO2max and changes in total blood volume (*r* = 0.65, *p* = 0.007), plasma volume (*r* = 0.58, *p* = 0.018) and red blood cell volume (*r* = 0.59, *p* = 0.015). Similarly, high correlations were noted between changes in vVO2max and changes in total blood volume (*r* = 0.55, *p* = 0.027), plasma volume (*r* = 0.54, *p* = 0.033) whereas the correlation between changes in vVO2max and changes in red blood cell volume did not reach statistical significance (*r* = 0.42, *p* = 0.097). No correlations were observed between alterations in performance in 5 × 1000 m and changes in the hematological parameters (Hbmass/intravascular volumes).

## Discussion

We tested the assumption that ischemic preconditioning applied prior to high-intensity interval training would enhance physiological adaptations and boost performance. Our hypothesis was that applying ischemic preconditioning before HIIT would increase running velocity improving training quality and in the long-term improving performance. Moreover, we assumed that the repetitive fashion of the ischemia-reperfusion maneuver would enhance hematological profile by abruptly trapping blood distal to the occlusion point and thus reducing venous return and central venous pressure. In turn, an increase in plasma and blood volume would be expected. In accordance with our hypothesis ISC group completed bouts of HIIT running at a higher velocity compared with CON and after 8-weeks VO_2_max and vVO_2_max was higher in ISC compared to CON. Moreover, ISC had more pronounced increases in blood and plasma volume after training. Finally, ISC had improved performance during the 5 × 1000 m running test.

The higher running velocity in the ISC group appears to have contributed to the higher VO_2_max and vVO_2_max and the improved time in running performance at the 5 × 1000 m trial observed at the post-training evaluation. A relatively high value of VO_2_max stands as a prerequisite for success in endurance performance events, so the higher VO_2_max found in ISC group after training reflects an enhanced potential for optimal performance (Jones and Kirby 2025; Unhjem 2024). An increased running velocity corresponding to VO_2_max (vVO_2_max) found in ISC group after training is also a parameter long-known to be a major determinant of endurance performance (Billat et al. 2001, 2003). Taken together, the higher VO_2_max and vVO_2_max in ISC after training probably explain the better performance recorded in the 5 × 1000 m field test. It is interesting that during HIIT sessions ISC individuals did not report higher RPE rates (compared to CON) despite reaching higher running velocities. As such, training combined with ischemic preconditioning was as much tolerable as regular training. Also, the ISC group completed HIIT in lower exercise times, and thus, had a significant decrease in weekly HIIT load (RPE·min). Finally, the RPE rates in our study are in accordance with previous studies suggesting that high-intensity aerobic intervals are executed at 16–17 of RPE rates (Seiler and Sjursen 2004; Seiler and Hetlelid 2005).

It is important to note, that even though ischemic preconditioning has been widely used for acute performance benefits in a single exercise bout or trial, some studies showed it also enhances performance in repeated trials (Patterson et al. 2015; Ferreira et al. 2016). As such, it could be used as an effective tool for HIIT just like in our study, boosting training workload and fortifying physiological adaptations.

Regarding probable mechanisms explaining the enhanced performance, we found that ischemic preconditioning compared to regular training elicited a higher degree of blood and plasma volume expansion. To our knowledge this is the first study demonstrating such an effect. Blood volume is correlated to VO_2_max (Schmidt and Prommer 2008). It is not clear whether increased blood and plasma volumes are due to ischemia application and subsequent hyperemia per se or because of the higher running velocity leading to higher training stimulus. It is known that endurance training leads to increases in plasma and blood volume in the first two weeks causing a decrease in hematocrit (Montero et al. 2017). An augmented plasma volume might further emerge in response to the ischemic preconditioning protocol provided that the muscle mass occluded is large enough. A possible mechanism might be that during occlusion, the trapped blood to the tissues below the cuff can possibly decrease venous return based on a compromised stroke volume (Cherouveim et al. 2023; Ozaki et al. 2010). If that was true, a decrease in central venous pressure would be expected which might stimulate an increase in plasma and blood volume through increases in fluid-regulating hormones as a previous study showed (Montero et al. 2016). Endurance training and ischemic preconditioning might act additively to augment plasma volume in the ISC group. Whatever the hypervolemic stimulus, it is expected that a fall in Hct leading to decreased oxygen supply in tissues such as kidneys works as a signal for erythropoiesis with increased EPO secretion leading to increases in red cell blood volume and hemoglobin mass, thus restoring Hct level in a homeostatic manner (Montero et al. 2017). Indeed, training in both groups increased red blood cell volume and HBmass confirming previous reports (Montero et al. 2017). The differential effect of our intervention on intravascular volumes (i.e. larger increases in total volume and plasma volume in the ISC group, similar increases in Hbmass and RCBV) should be further investigated since the duration of the protocol was deemed sufficient to allow for hematological adaptations. However, an enhanced blood and plasma volume could have induced independently of the ischemia-reperfusion maneuver per se, but from the subsequent enhanced running velocity during HIIT, augmenting training quality and finally fortifying the training stimulus. Such an effect could have involved the kidneys and related fluid retention mechanisms that were caused by higher sympathetic activation originated from the increased power output resulting in higher renal vasoconstriction and decreased GFR (glomerular filtration rate) (Siebenmann et al. 2017a, b). Alternatively, a higher secretion of renin could upregulate the function of the angiotensin-aldosterone pathway (Siebenmann et al. 2017a, b). The higher systolic blood pressure at peak exercise in ISC group found in post-training period in the present study may lend support to this hypothesis. Whatever the case, the finding of a more pronounced increase for blood and plasma volume in ISC group is quite innovative and promising for future research.

Another interesting finding is the contrasting outcome in blood pressure between groups. Even though there was no difference from pre to post values of systolic and mean blood pressures for both groups, there was a significant interaction, as peak systolic blood pressure slightly fell after training in CON but increased in ISC. Previous studies support a slight fall in blood pressure after training both at rest and exercise (Cornelissen and Fagard 2005; Cornelissen et al. 2010) just as CON group did in our study, but the raise in ISC group consists new evidence and may reflect the higher running velocity leading to increased cardiovascular response or the fluid retention by the kidneys as previously mentioned. Recent evidence supposes that a higher blood pressure can be ergogenic leading to increased perfusion and blood supply in tissues as long as functional sympatholysis is not impaired (Calbet and Joyner 2010). If that is true, the increased peak systolic blood pressure in ISC group could have contributed to performance enhancement.

It should be mentioned though, that enhanced performance could be also induced independently from blood volumes changes, by cardiac remodeling and increased left ventricular dimensions following interval training resulting in increases in cardiac output (Eriksson et al. 2024). In addition, ischemic preconditioning enhances neural activation augmenting muscle force of the exercising muscles leading to an enhanced capacity to produce more power (Cruz et al. 2024). In our study, the latter can explain the enhanced running velocity of the ISC group in the HIIT boosting training performance, and in the long-term could have contributed to the enhancement of physiological adaptations and finally, running performance. It shoud be mentionted that the latter could have led to improvements in peripheral adaptations such as increased mitochondria and oxygen extraction (Skattebo et al. 2020). Last, the maneuver of ischemia-reperfusion may lead to enhanced activation of Nrf2, a key transcriptor factor that regulates various biological processes like redox balance, mitochondrial biogenesis and antioxidant capacity, which in turn could improve performance (Martinez-Canton et al. 2024). Collectively, the higher running velocity as a training stimulus in the ISC group could trigger various adaptive mechanisms such as enhanced cardiac output, increased muscle force, augment oxygen extraction and mitochondria biogenesis; leading to higher VO_2_max and performance in this training group.

Regarding the fact that we chose to perform a cycling exercise test even that this activity does not match to running training according to our participant’s main activity, that was to measure blood pressure during exercise in which the participants must remain seated. A previous study with runners and running training also used a cycling exercise test to measure oxygenation responses and found positive outcomes after training, meaning that cycling exercise can be reliable and sensitive to detect physiological adaptations even after running training (Park et al. 2019). Therefore, the results of the present study regarding the maximal values between the two tests (running and cycling incremental tests) show a similar and comparable physiological response. It is well-accepted that in cycling exercise there is a lower VO_2_max compared to running due to the smaller muscle mass recruited, which is evident in our results. However, carbon dioxide production, respiratory exchange ratio, ventilatory equivalents, end-tidal partial pressure for oxygen and carbon dioxide were similar between the two forms of exercise and can indicate the validity of cycling usage to assess physiological variables after running training in future studies.

**Table 1:** Mean±SD values of physiological traits in control (*n*=8) and ischemia (*n*=8) group before and after 8 weeks of training

	CON		ISC		Effect of time	Interaction
	Pre	Post	Pre	Post	*p*	*p*
Age (yrs)	34.8±5.4		33.4±4.9			
Height (cm)	175±3		178±4			
Weight (kg)	70.5±4.9	69.5±4.5	71.2±3.3	70.0±3.2	0.574	0.771
Fat (%)	11.0±3	10.2±3	11.1±3	9.8±3	0.775	0.861
BMI (kg/m^2^)	23.0±3.5	22.7±3.3	22.5±3.1	22.1±3.0	0.796	0.832
Hct (%)	44.9±0.7	46.4±1.3	44.0±2.6	45.2±1.0	0.102	0.087
[Hb] (g/dl)	15.8±1.2	15.6±1.2	16.2±1.1	14.8±0.8*^§^	0.023	0.044

*BMI* body mass index; *Hct* hematocrit; *[Hb]* hemoglobin concentration

*Significant difference pre vs. post in ISC, *p*<0.05

^§^Significant difference between groups in post. *p*<0.05

**Table 2:** Mean±SD values of training characteristics in control (*n*=8) and ischemia (*n*=8) group during 8 weeks of training

	CON	ISC	*p*
Weekly running training volume (km)	80.6±4.0	80.4±3.0	0.845
Weekly continuous training volume (km)	66.3±4.0	66.1±3.0	0.879
Continuous training heart rate (bpm)	156±12	152±11	0.436
Continuous training RPE (6-20)	11.2±1.5	10.7±0.9	0.196
Total number of intervals	16	16	
Weekly interval training volume (km)	14.3±1.0	14.3±1.0	
Weekly interval training volume (min)	50±3	48±4	0.115
Total interval training volume (km)	114±8	114±8	
Total interval training volume (min)	402±24	389±32*	0.001
Interval trainining average speed (km/h)	17.4±0.3	18.0±0.4*	0.001
Interval training heart rate (bpm)	176±7	178±8	0.769
Interval training RPE (6-20)	16.7±0.6	16.5±0.6	0.512
Weekly interval training load (RPE·min)	835±77	802±82*	<0.001

*RPE* rate of perceived exertion

*Significant difference between groups *p*<0.01

**Table 3:** Mean±SD values of physiological parameters of the incremental running exercise test in control (*n*=8) and ischemia (*n*=8) group before and after 8 weeks of training

	CON		ISC		Effect of time	Interaction
	Pre	Post	Pre	Post	*p*	*P*
vVO_2_max (km/h)	18.2±0.7	18.9±0.6*	18.4±0.9	19.6±0.9*^§^	<0.001	0.048
VE (L/min)	157.9±13.8	161.2±12.6	161.8±17.2	169±19.0	0.154	0.798
RER	1.28±0.11	1.24±0.13	1.30±0.10	1.25±0.12	0.604	0.521
RR (breath/min)	57±4	56±4	55±5	55±4	0.876	0.797
VE/VO_2_	40.1±2.1	40.1±2.4	41.3±2.3	40.1±2.6	0.687	0.172
VE/VCO_2_	31.3±2.1	32.1±2.4	31.7±2.3	31.9±2.6	0.105	0.581
PETO_2_ (mmHg)	114.0±3.0	113.8±3.6	110.5±2.9	111.6±3.4	0.583	0.467
PETCO_2_ (mmHg)	37.6±2.5	37.3±2.8	39.1±3.1	38.0±3.2	0.455	0.675
vVT_2_ (km/h)	13.8±1.2	14.3±0.9*	13.9±0.9	14.4±1.1*	<0.001	0.987
VO_2_ VT_2_ (ml/kg/min)	42.4±4.4	44.4±4.3*	43.6±2.7	46.6±3.7*	<0.001	0.391
VO_2_ VT_2_ (L/min)	2.98±0.25	3.08±0.31	3.10±0.29	3.26±0.34	0.061	0.089
%VO_2_max	77.8±6.3	79.2±5.0*	78.3±3.6	81.8±2.3*	0.004	0.901
HR max (bpm)	185±7	184±7	185±5	185±6	0.956	0.798
HR VT_2_ (bpm)	160±6	159±9	165±6	164±6	0.853	0.753

*vVO*_*2*_*max* maximal aerobic velocity; *VE* ventilation; *RER* respiratory exchange ratio; *RR* respiratory ratio; *PETO*_*2*_ end-tidal partial pressure of oxygen; *PETCO*_*2*_ end-tidal partial pressure of carbon dioxide; *vVT*_*2*_ velocity at second ventilatory threshold; *VO*_*2*_ *vVT*_*2*_ oxygen consumption at second ventilatory threshold; *HR max* maximal heart rate; *HR VT*_*2*_ heart rate at second ventilatory threshold

*Significant difference between pre vs. post

^§^Significant difference between groups in post. *p*<0.05

**Table 4:** Mean±SD values of physiological parameters of the incremental cycling exercise test in control (*n*=8) and ischemia (*n*=8) group before and after 8 weeks of training

	CON		ISC		Effect of time	Interaction
	Pre	Post	Pre	Post	*p*	*p*
PPO (watts)	295±8	306±9*	300±10	315±11*§	0.005	0.031
VO_2_max (L/min)	3.59±0.22	3.75±0.28*	3.63±0.31	3.82±0.35*	0.003	0.437
VO_2_max (ml/kg/min)	51.0±2.1	54.0±1.7*	51.2±1.5	54.6±1.9*	<0.001	0.486
VE (L/min)	132.6±16.2	139.0±19.9	137.4±14.3	143.0±18.8	0.236	0.532
RER	1.31±0.04	1.29±0.05	1.30±0.04	1.28±0.03	0.587	0.497
RR (breath/min)	56±3	57±2	55±4	56±4	0.786	0.803
VE/VO_2_	36.9±2.3	37.0±2.9	37.8±2.1	37.4±2.5	0.723	0.587
VE/VCO_2_	28.2±2.0	28.7±2.2	29.1±2.4	29.3±2.7	0.574	0.611
PETO_2_ (mmHg)	113.1±3.1	113.6±3.2	112.3±3.0	112.5±3.5	0.781	0.677
PETCO_2_ (mmHg)	37.6±2.5	37.3±2.8	39.1±3.1	38.0±3.2	0.455	0.675
Watt VT_2_ (watts)	206±12	214±16	210±11	218±19	0.341	0.653
VO_2_ VT_2_ (ml/kg/min)	35.7±2.4	37.3±3.2*	35.9±2.2	38.3±2.9*	<0.001	0.118
VO_2_ VT_2_ (L/min)	2.51±0.20	2.60±0.25*	2.54±0.22	2.68±0.28*	0.041	0.167
%VO_2_max	70.1±3.3	70.3±3.1	70.3±2.6	70.3±2.9	0.783	0.823
HR max (bpm)	183±6	183±5	182±3	181±4	0.816	0.801
HR VT_2_ (bpm)	157±6	156±7	158±4	155±7	0.645	0.512

*PPO* peak power output; *VO*_*2*_*max* maximal oxygen consumption; *VE* ventilation; *RER* respiratory exchange ratio; *RR* respiratory ratio; *PETO*_*2*_ end-tidal partial pressure of oxygen; *PETCO*_*2*_ end-tidal partial pressure of carbon dioxide; *Watt VT*_*2*_ power at second ventilatory threshold; *VO*_*2*_ *vVT*_*2*_ oxygen consumption at second ventilatory threshold; *HR max* maximal heart rate; *HR VT*_*2*_ heart rate at second ventilatory threshold

*Significant difference pre vs. post within groups

^§^Significant difference between groups in post. *p*<0.05

### Limitations

Potential limitations in the lay out of the study have to be conceded. Measuring blood pressure with Finapres device during exercise of high-intensity may overestimate true blood pressure values. Thus, interpreting the absolute values of this study of blood pressure needs caution. Comparing relative systolic blood pressures differences among experimental conditions in the present study still keep its value even though finger artifacts might intervene with vasodilation after ischemia-reperfusion training in a different way than in regular training. Secondly, for hematological variables, given the multitude of scheduled exercise trials, the findings of the present study seem constrained by virtue of single assessments of hemoglobin mass being performed in our participants. Consecutive triplicate measurements have been conducted in recent studies and elevated COHb to previously considered harmful levels (Krumm et al. 2024). Not only would a duplicate measurement of hemoglobin mass have reduced the typical error of the measurement by 1.4% but also the related CO dosage (and the resultant COHb formation) would not constitute a health concern. The fact that we resorted to pulse CO-oximetry for the non-invasive determination of COHb (SpCO) stands as a limiting factor of the precision of our assessments. Fagoni et al. (2018) have recently demonstrated a close agreement between ΔSpCO and ΔCOHb when hemoglobin mass and total blood volumes are not that high as is the case with our participants (see Fig. 3). As far as the determination of ΔCOHb is concerned, the use of a blood gas analyzer to provide COHb values in duplicate blood samples seems warranted in future studies. Last, even though we used the application of cuffs in the CON group with minimal pressure to counteract the placebo effect, we cannot exclude it as a factor interfering in our results. The high pressure of the cuffs in ISC group produces different sensory cues as limb color change, pain, and reperfusion relief, cues that were not elicited in the CON group. Moreover, ischemia-reperfusion maneuver could produce expectations to the participants or subsequent increased motivation specially when training and performance sessions are followed. Except from the placebo effect, ischemia could induce nocebo effect from the pain of the pressure cuff during occlusion and the blood flow restriction and lead to negative experiences. Finally, the placebo and nocebo effects are difficult to be controlled and could have influenced the study’s outcome, especially when performance is assessed. However, participants in a group did not know the experimental condition of the other group, so participants in CON did not know the hypothesis and the effects of occlusion. Moreover, before the termination of the study, information about the effects of ischemic preconditioning on performance was not conveyed to anybody, so as, not to produce expectations to the participants.

## Conclusion

Finally, training combined with ischemic preconditioning increased running velocities during HIIT sessions and after 8-weeks resulted in more pronounced increases in VO_2_max and vVO_2_max in ISC compared to CON. Moreover, the long-term application of ischemia-reperfusion in ISC potentiated, either directly or indirectly, the higher blood and plasma volume increase in response to training. The above parameters, all major determinants of endurance performance, could have contributed to the performance enhancement as reflected in improved running times in ISC compared to CON in the specific field test (5 × 1000 m). Thus, ischemic preconditioning increasing running velocity in HIIT could be used as an effective tool to optimize performance.

## Supplementary Information

Below is the link to the electronic supplementary material.

Supplementary Material 1
