# Supplementary material for: Applying ischemic preconditioning prior to endurance training improves hematological profile and performance in long-distance runners
Source: Eur J Appl Physiol. 2026 Jan 16;126(5):2681–93. doi: 10.1007/s00421-025-06120-6 (PMC13236773; doi:10.1007/s00421-025-06120-6)
Supplement: Supplementary file 1 — Supplementary Material 1 [file 421_2025_6120_MOESM1_ESM.docx]

|  | **Monday** | **Tuesday** | **Thursday** | **Friday** | **Sunday** |
| --- | --- | --- | --- | --- | --- |
| **WEEK 1** | **10km 70-80% vVO_2_max** | **15x200m 100% vVO_2_max/ 1:1 rest** | **12km 70-80% vVO_2_max** | **5x1000m 95% vVO_2_max/ 2 min rest** | **16km 70-80% vVO_2_max** |
| **WEEK 2** | **10km 70-80% vVO_2_max** | **10x300m 100% vVO_2_max/ 1:1 rest** | **12km 70-80% vVO_2_max** | **4x2000m 92% vVO_2_max/ 3 min rest** | **20km 70-80% vVO_2_max** |
| **WEEK 3** | **10km 70-80% vVO_2_max** | **8x400m 100% vVO_2_max/ 1:1 rest** | **12km 70-80% vVO_2_max** | **6x800m 97% vVO_2_max/ 2 min rest** | **25km 70-80% vVO_2_max** |
| **WEEK 4** | **10km 70-80% vVO_2_max** | **6x600m 98% vVO_2_max/ 1:1 rest** | **12km 70-80% vVO_2_max** | **3x3000m 90% vVO_2_max/ 3 min rest** | **30km 70-80% vVO_2_max** |
| **WEEK 5** | **10km 70-80% vVO_2_max** | **15x200m 100% vVO_2_max/ 1:1 rest** | **12km 70-80% vVO_2_max** | **5x1000m 95% vVO_2_max/ 2 min rest** | **16km 70-80% vVO_2_max** |
| **WEEK 6** | **10km 70-80% vVO_2_max** | **10x300m 100% vVO_2_max/ 1:1 rest** | **12km 70-80% vVO_2_max** | **4x2000m 92% vVO_2_max/ 3 min rest** | **20km 70-80% vVO_2_max** |
| **WEEK 7** | **10km 70-80% vVO_2_max** | **8x400m 100% vVO_2_max/ 1:1 rest** | **12km 70-80% vVO_2_max** | **6x800m 97% vVO_2_max/ 2 min rest** | **25km 70-80% vVO_2_max** |
| **WEEK 8** | **10km 70-80% vVO_2_max** | **6x600m 98% vVO_2_max/ 1:1 rest** | **12km 70-80% vVO_2_max** | **3x3000m 90% vVO_2_max/ 3 min rest** | **16km 70-80% vVO_2_max** |

**Training intervention in CON (n=8) and ISC (n=8) groups.**
